# S99. CANNABIS USE, PSYCHOTIC-LIKE EXPERIENCES AND ABERRANT SALIENCE IN A SAMPLE OF BELGIAN STUDENTS

**DOI:** 10.1093/schbul/sby018.886

**Published:** 2018-04-01

**Authors:** Francesco Bernardini, Chiara Gobbicchi, Luigi Attademo, Severin Puchalski, Patrizia Moretti, Alfonso Tortorella, Gwenole Loas

**Affiliations:** 1 Free University of Brussels; 2 Università degli Studi di Perugia; 3 ASP Basilicata; 4 Erasme Hospital

## Abstract

**Background:**

Cannabis is the most popular illicit drug in the western world and its use seems to be strongly associated with an increased risk of developing schizophrenia and other psychotic disorders. Its use can induce transient psychotic symptoms in healthy individuals and increase rate of subclinical psychotic symptoms in the general population. Subclinical psychotic experiences (also called Psychotic Like Experiences: PLEs), such as magical thinking, paranoid ideation or hallucinations, could be considered as a phenotype qualitatively similar to the symptomatology of psychotic disorders but quantitatively less severe in terms of intensity, frequency and impairment. They are fairly common in the general population and usually transitory and self-limiting but they could become abnormally persistent and evolve to a full-blown psychotic disorder, especially if combined with certain environmental risk factors, such as trauma, urbanicity, cannabis use. PLEs may be considered as an early marker of a latent psychosis vulnerability and the frequently good outcome of subclinical psychosis can be turned in negative outcomes by the association with environmental risk factors, such as cannabis use. We focus our attention on aberrant salience, a peculiar psychotic experience, frequently reported during the prodromal phase that precede the onset of full-blown psychotic illness. Aberrant salience is the unusual or incorrect assignment of salience or significance to innocuous stimuli; it has been hypothesized to be an important mechanism in the development of psychosis.

**Methods:**

Undergraduate students of ULB (Universitè Libre de Bruxelles) and INSAS (Institut national supérieur des arts du spectacle) of Brussels (Belgium) were invited to participate to the study.

A self-report questionnaire, investigating socio-demographic characteristics, and cannabis use was administered, evaluating lifetime and current cannabis use.

Aberrant Salience Inventory (ASI) is a 29 item Yes–No questionnaire developed to evaluate aberrant salience. French version of Community Assessment of Psychic Experiences (CAPE), was used to evaluate dimensions of psychosis and PLEs. CAPE is a 42-item, self-report questionnaire, developed to measure the lifetime prevalence of PLEs in the general population. The questionnaire assesses three symptom dimensions (positive, depressive and negative symptoms). All statistical analysis was carried out with Statistical Package for Social Sciences, Version 20.0. We evaluated individual correlations between years of cannabis use and days of cannabis use in the last month with the tools scores. We also explored correlation of ASI score with different CAPE scores and different types of PLEs. Correlations were carried out by using the nonparametric Spearman correlation test.

**Results:**

The final sample was of 257 participants. 46,3% of subjects reported a lifetime cannabis use and 35.0% reported a current cannabis use (last 30 days). Compared with non-users, cannabis users showed significant higher ASI scores and also higher positive and negative dimensions CAPE scores. No significant association was found between cannabis consumption and the depressive dimension of CAPE. Years of cannabis use and frequency of use in the last 30 days showed a small positive correlation with ASI score; also, weaker positive correlations with CAPE positive and negative dimensions scores were observed.

**Discussion:**

To some extent, our results support the evidences that cannabis use is associated with an increased rate of psychotic experiences in individuals without clinical form of psychosis. Future prospective longitudinal studies are required to better investigate the meaning of the association between cannabis use and PLEs.

